# Effect of Task Constraints During Drop Vertical Jump Test on Movement Variability in Anterior Cruciate Ligament Reconstructed Female Basketball Players: A Nonlinear Approach

**DOI:** 10.1002/ejsc.70185

**Published:** 2026-07-20

**Authors:** Sara González‐Millán, Toni Caparrós, Víctor Toro‐Román, Carla Pérez‐Chirinos Buxadé, Víctor Illera‐Domínguez, Lluís Albesa‐Albiol, Gerard Moras, Bruno Fernández‐Valdés

**Affiliations:** ^1^ Department of Health Sciences Research Group in Technology Applied to High Performance and Health TecnoCampus Universitat Pompeu Fabra Barcelona Spain; ^2^ University of San Jorge Zaragoza Spain; ^3^ Research Group on Physical Activity, Nutrition and Health (GRAFAiS) National Institute of Physical Education of Catalonia (INEFC) University of Barcelona Barcelona Spain; ^4^ Sport Research Institute Universitat Autònoma de Barcelona Barcelona Spain

**Keywords:** drop jump, dual task, entropy, injury, knee

## Abstract

The aim of this study was to analyze the impact of an Anterior Cruciate Ligament (ACL) injury on movement variability (MV) and biomechanical variables in female basketball players during a drop vertical jump (DVJ) task, both with and without a ball. Female basketball players (*n* = 15) participated in this study. The participants performed two jump in each of six conditions (total = 12 jumps) in randomizer order: (i) Bilateral No Ball (2NB)—two‐leg jump without a ball; (ii) Bilateral Ball (2B)—two‐leg jump with a ball; (iii) ACL No Ball (ACLNB)—injured‐leg jump without a ball; (iv) ACL Ball (ACLB)—injured‐leg jump with a ball; (v) No ACL No Ball (NOACLNB) –uninjured‐leg jump without a ball; (vi) No ACL Ball (NOACLB)—uninjured‐leg jump with a ball. DVJ performance parameters were assessed using an accelerometer placed on the lower back and a force platform. MV was quantified using the multiscale entropy (MSE) derived from the acceleration data. The Complexity Index (CI) was also calculated. The ACLB condition exhibited the highest sample entropy (SampEn) value across all scales, followed by the 2B and NOACLB conditions, whereas ACLNB showed the lowest values. The CI also indicated significant variability across conditions. For linear biomechanical variables, significant differences were only observed in contact time (CT) between NOACLB and NOACLNB. The inclusion of a task constraint did not result in differences between ACL and NOACL groups in linear jumping performance metrics. However, nonlinear MV analyses, using MSE and CI, revealed condition differences.

## Highlights

- The inclusion of a task constraint did not lead to significant differences in linear jumping performance variables between Anterior Cruciate Ligament (ACL) and NOACL limbs.
- Nonlinear Movement Variability (MV) analyses, using Multiscale entropy (MSE) and Complexity Index (CI), revealed lower entropy and complexity in the no‐ball condition and higher values when a task constraint (ball) was introduced.
- Despite the recovery of physical performance, the previously injured leg displayed a more rigid movement strategy under low task demands. When task constraints increase, neuromuscular adaptability deficits become evident.

## 1 Introduction

Basketball is a team sport that involves the complex interplay of tactical, technical, psychological, and physiological components (Narazaki et al. 2009). Repeated jumps, sprints, accelerations, decelerations, changes of direction, and specific movements (shooting, rebounding, and dribbling…) are fundamental determinants of basketball performance (Gottlieb et al. 2021; Petway et al. 2020). These physical demands are essential for achieving high‐level performance; however, they are also associated with an increased risk of injury (Andreoli et al. 2018).

The lower limbs are the most frequently injured body region in basketball (Starkey 2000; Torres‐Ronda et al. 2022), with the ankle and knee accounting for the majority of reported cases (Andreoli et al. 2018; Drakos et al. 2010; Starkey 2000). Sex‐specific differences have also been reported. Andreoli et al. (2018) found that 19.5% of injuries in female players occurred in the ankle, while 20.6% affected the knee. In contrast, male players exhibited lower injury rates, with 14.6% affecting the ankle and 17.5% affecting the knee.

Among knee injuries, anterior cruciate ligament (ACL) injuries are particularly concerning in professional basketball due to their high incidence, severity, and prolonged recovery times (Stojanović et al. 2023). In the National Basketball Association (NBA), ACL injuries have a reported incidence of approximately 2.5 cases per year, resulting in prolonged periods of inactivity (∼10 months) (Starkey 2000). Tosarelli et al. (2024) found that ACL injuries in professional basketball typically occur during offensive actions when the player possesses the ball and is closely guarded by a defender. Specifically, 47.0% of ACL injuries resulted from offensive cutting maneuvers, while 22.0% occurred during jump landings (Tosarelli et al. 2024).

Return‐to‐play (RTP) assessments are critical in the rehabilitation process following ACL injuries, particularly in sports such as basketball where high‐intensity movements are integral to performance (Gottlieb et al. 2021; Petway et al. 2020). Among the various functional tests used in RTP protocols, jump tests are frequently included due to the central role of jumping ability in key basketball‐specific actions such as shooting, rebounding, dunking, layups, shot‐blocking, and defensive movements (Engelen‐van Melick et al. 2013; Häkkinen 1991; Yayra Kwaku Ashigbi et al. 2020). However, conventional jump assessments often involve pre‐planned, predictable tasks that fail to capture the reactive and unpredictable nature of in‐game movement demands (Travassos et al. 2011; Wilk et al. 2023). This discrepancy may lead to premature clearance for competition, as athletes might not be adequately prepared for the coordinative demands of real‐game scenarios, thereby increasing their risk of re‐injury (Wilk et al. 2023).

Human movement emerges from the coordinated interaction of multiple biological systems and follows complex, nonlinear dynamics (Goldberger et al. 1990; Naranjo Orellana and De La Cruz Torres 2010). Despite this, linear methodologies have traditionally dominated the analysis of human health and movement, although they are limited in their ability to describe biological variability (Stergiou and Decker 2011; Naranjo Orellana and De La Cruz Torres 2010). To overcome these limitations, nonlinear analytical methods may offer a more comprehensive understanding of motor variability, adaptability, and neuromuscular function. Traditionally, jump performance has been evaluated using linear biomechanical variables such as jump height or distance, CT, and the reactive strength index (RSI) (Pleša et al. 2022). While these metrics provide insight into physical performance, they do not fully account for differences in movement strategies or neuromuscular adaptations following injury. For instance, athletes may achieve similar jump height or jump distance through distinct biomechanical strategies, leading to potential compensatory patterns that are overlooked in linear analyses (Bishop et al. 2023; Srinivasan et al. 2018).

Traditionally, ACL rehabilitation has been approached from a linear perspective, focusing on stability and movement control as primary objectives. However, it has been suggested that effective recovery should incorporate movement variability (MV) and recognize neuromuscular dynamics from a complex systems perspective (Bittencourt et al. 2016). MV, assessed through entropy measures and fractal analysis, has emerged as a key indicator of motor adaptability following injury (Chaney et al. 2023; Hollman et al. 2021, 2022).

Multiscale Entropy (MSE) analysis is a nonlinear method that quantifies the complexity of motor system output across multiple time scales, offering a more comprehensive view of movement adaptability compared to traditional entropy measures like Sample Entropy (SampEn) or Approximate Entropy (ApEn) (Busa and van Emmerik 2016). By evaluating how signal regularity evolves with scale, MSE allows detection of patterns associated with motor rigidity or excessive variability under different task demands. The derived Complexity Index (CI), calculated as the area under the MSE curve, serves as a global marker of system adaptability, with higher values indicating greater neuromuscular flexibility and lower values reflecting loss of complexity due to aging, fatigue, or injury (Costa et al. 2002). These results align with the Loss of Complexity Hypothesis, which states that biological systems under stress or dysfunction exhibit reduced variability and adaptability (Lipsitz et al. 1992). In sports science, MSE and CI have been validated as sensitive tools for detecting neuromuscular deficits, with studies showing that athletes exposed to task‐specific constraints (e.g., unstable surfaces or cognitive loads) exhibit higher complexity metrics, likely reflecting enhanced adaptability and reduced injury risk (Exel et al. 2019; Moras et al. 2018). A healthy neuromuscular system maintains an optimal level of MV, enabling efficient adjustments to external perturbations. In contrast, individuals with a history of ACL injury exhibit reduced entropy in joint kinematics, suggesting increased rigidity and a lower adaptive capacity, which could elevate the risk of reinjury (Decker et al. 2011; Quirino et al. 2024; Zampeli et al. 2010). Despite recovery in linear performance parameters, increased regularity in movement patterns does not necessarily reflect successful recovery, but may instead signal reduced system flexibility in response to dynamic environmental demands (van de Ven et al. 2023).

The aim of this study was to analyze the impact of ACL injury on MV and linear biomechanical variables in female basketball players during a drop vertical jump (DVJ) task, performed with and without a ball. We hypothesize that (i) players with a history of ACL injury will exhibit lower MV and CI values in the injured leg compared to the uninjured leg, indicating greater rigidity despite restored physical performance; (ii) athletes with ACL injuries will exhibit lower jump height and longer CT due to compensatory strategies; and (iii) the inclusion of the ball in the DVJ task will increase MV and CI in lower limbs due to greater cognitive and coordinative demands.

## 2 Materials and Methods

### 2.1 Participants

A total of 18 female basketball players from different clubs in the Barcelona province (Spain) were recruited for this study. However, three players were excluded due to inertial measurement unit (IMU) signal recording failures, resulting in signal loss that rendered data analysis unreliable. Consequently, the final sample consisted of 15 players (age: 22.33 ± 5.34 years; body mass: 67.35 ± 10.37 kg; height: 1.69 ± 0.05 m; experience: 14.20 ± 4.55 years; weekly training: 7.51 ± 1.75 h; 93.33% were right‐leg dominant).

The sample size of 15 participants corresponds to a statistical power of 0.938 for detecting a moderate‐to‐large effect size (Cohen's *d* = 0.89) using a two‐tailed paired Wilcoxon signed‐rank test, with an alpha level of 0.05 (GPower v3.1). However, it is important to consider the specific characteristics of this sample, as all participants had a history of ACL injury in only one limb and had been medically cleared for full sports participation at the time of the study.

All participants had undergone at least one ACL reconstruction surgery on either their left limb (*n* = 4) or right limb (*n* = 11) at least 1 year prior to the present study. The surgical details are summarized in Table 1. The ACL reconstructions involved different graft types, including allografts (*n* = 2), bone‐patellar tendon‐bone autografts (*n* = 2), semitendinosus tendon autografts (*n* = 10), and one unspecified ACL reconstruction (*n* = 1). All players had received medical clearance to return to full sports participation at least 1 month before undergoing the study assessments.

**TABLE 1:** Characteristics of participants' surgeries.

Participants	ACL surgeries (*n*°)	Type of surgery^a^	Time elapsed since last injury to last surgery (months)	Time elapsed since last surgery to DVJ test (months)	Time since last surgery to return to competition (months)
1	1	2	1	25	16
2	2	1	4	24	15
3	1	1	3	16	5
4	2	4	1	16	8
5	1	1	1.5	24	11
6	1	3	3	19	4
7	1	1	1	96	86
8	3	1	5	15	2
9	1	2	5	96	89
10	4	1	2.5	17	3
13	1	1	1	59	42
14	1	1	2	26	13
16	1	1	14	13	1
17	1	2	15	53	40
18	1	2	2	28	18

^a^ 1: ACL; 2: ACL + Internal Meniscus; 3: ACL + Externa Meniscus; 4: Partial ACL + Internal Meniscus; DVJ: drop vertical jump.

The inclusion criteria for participation in the study were: (i) female basketball players over 16 years of age with an active federated license; (ii) active competition until the 2023–2024 season in categories above the sixth national division; (iii) a history of one or more ACL surgeries (total or partial) in one leg while having a healthy, non‐operated contralateral leg; (iv) absence of acute musculoskeletal or joint injuries, as well as no discomfort on the day of assessment or within the previous seven days; (v) a regular training schedule (at least three sessions per week); (vi) return to competition at least one month before data collection; and (vii) signed informed consent.

Eligibility was assessed through a pre‐study health history questionnaire. The exclusion criteria included: (i) history of bilateral ACL surgery; (ii) lack of medical clearance for sports participation; and (iii) inability to complete the assessments due to fear or pain.

The study protocol was reviewed and approved by the Ethics Committee for Clinical Research of the Catalan Sports Council (026/CEICGC/2021). All athletes were informed about the study's purpose and provided written informed consent.

### 2.2 Study Design

This study was designed as an analytical and experimental investigation, based on a previous published protocol (González‐Millán et al. 2024). Each participant completed two sessions (separated by 24 h): a familiarization session and an evaluation session.

Athletes performed DVJ using either one (Unilateral) or both (Bilateral) lower limbs, under different conditions: (i) Bilateral No Ball (2NB)—two‐leg jump without a ball; (ii) Bilateral Ball (2B)—two‐leg jump with a ball; (iii) ACL No Ball (ACLNB)—injured‐leg jump without a ball; (iv) ACL Ball (ACLB)—injured‐leg jump with a ball; v) No ACL No Ball (NOACLNB) –uninjured‐leg jump without a ball; (vi) No ACL Ball (NOACLB)—uninjured‐leg jump with a ball. All assessments were conducted indoors before team training sessions. During both the familiarization and evaluation sessions, athletes performed two repetitions per condition, resulting in a total of 12 jumps per session (2 repetitions × 6 conditions). The order of conditions was randomized, and a 60‐s rest interval was provided between jumps. No verbal feedback regarding movement quality or test results was given to participants. A standardized warm‐up was performed before testing, consisting of 5 minutes of self‐paced cycling and dynamic stretching exercises, resembling a typical pre‐game warm‐up routine. All DVJ tests were performed under similar environmental conditions (20°C–25°C and 65%–75% humidity) at the same time of day (± 1 h).

### 2.3 Drop Vertical Jump Test

Participants performed DVJ from a 30‐cm‐high fixed bench, following the protocol described by González‐Millán et al. (2024). They began in an upright position with their hands on their hips. Upon instruction, they were required to drop vertically, land on the force plate, and execute a maximal vertical jump as quickly as possible after ground contact. For unilateral jump conditions, players were required to land and jump using the specified limb.

### 2.4 Dual‐Task Condition

The dual‐task condition involved performing the DVJ while catching a ball in mid‐air (González‐Millán et al. 2024). A trained research assistant, who remained consistent throughout the study, delivered a frontal chest pass from 3 m. Participants were instructed to initiate their movement as soon as the ball left the passer's hands. Participants started with hands on hips and, after the DVJ started, were required to catch the ball mid‐air during the DVJ execution, and land on the force platform while holding it. This task was performed for the ball‐included conditions: 2B, NOACLB, and ACLB.

### 2.5 Equipment

A force platform and IMU were used for data collection.

The force platform (MuscleLab, Ergotest Technology AS, Langesund, Norway) recorded data at a sampling rate of 1000 Hz. It consisted of four strain gauge sensors with a total force capacity of 20 kN. The force platform was connected to a computer via MuscleLab V8.27 software, which recorded linear parameters performance: jump height, contact time (CT), and RSI. Jump height was computed using the impulse‐momentum theorem (Kirby et al., 2011). A WIMU IMU device (Realtrack Systems, Almería, Spain; mass: 70 g; dimensions: 81 × 45 × 16 mm) was used to record acceleration data at 1000 Hz. The IMU was secured at the L4‐L5 spinal level using an adjustable sports belt, as this placement provides the most accurate representation of whole‐body movement (Montgomery et al. 2010).

### 2.6 Data Analysis

For analysis, given the maximal nature of vertical jump tests, previous research recommends using the highest jump from multiple trials (Moir et al. 2008). Therefore, the highest jump from the two attempts was selected. Raw acceleration data from all three axes (vertical, mediolateral, anteroposterior) were extracted using SPRO Software v1.0.0 (Realtrack Systems, Spain). The total acceleration (AcelT), originally recorded at 1000 Hz and subsequently resampled at 400 Hz, was computed as the vector sum of the three components, following the methodology of Gómez‐Carmona et al. (2020) and Moras et al. (2018).

Cuts were applied to the acceleration signal for each repetition of the DVJ from the descent from the bench to the second landing. The phases composing the DVJ were defined as (Harry et al. 2024): (i) bench descent; (ii) initial landing; (iii) maximum vertical jump; and (iv) second landing. The complete signal was divided into homogeneous segments corresponding to ball and non‐ball conditions, and heterogeneous portions were removed. The segment length was sufficient to ensure SampEn reliability. The values for the sequence length (*m*), tolerance (*r*) and data length (*N*) were as follows: *m* = 2, *r* = 0.2x standard deviation, and N was greater than 1000 data points.

MSE analysis was used to quantify the regularity of AcelT signals across multiple time scales (nonlinear analysis) The MSE algorithm integrates a coarse‐graining process with the SampEn algorithm to assess entropy at various time scales, offering insights into fluctuations in movement patterns (Goldberger et al. 2000). The CI was calculated as the area under the MSE curve, providing a global measure of movement complexity (Busa and van Emmerik 2016).

### 2.7 Statistical Analysis

Statistical analyses were conducted using R (v4.2.2, R Foundation for Statistical Computing, Vienna, Austria). Data for each participant, along with mean values, were visualized using violin plots. The Shapiro‐Wilk test was employed to assess normality, and Levene's test was used to evaluate the homogeneity of variances.

Due to the non‐normal distribution of some parameters in the DVJ, nonparametric tests were applied. The Friedman test was used to compare DVJ conditions, and post hoc comparisons were performed using Wilcoxon signed‐rank tests. *p*‐values were not adjusted for multiple comparisons (e.g., Bonferroni) due to the exploratory nature of the study and the small sample size. Therefore, results should be interpreted with caution. The significance level was set at *p* < 0.05.

Comparisons were also assessed using Cliff's delta (*δ*), a nonparametric effect size measure suitable for data that does not meet normality assumptions. Effect size magnitudes were interpreted as follows: |δ| < 0.147, trivial; 0.147–0.33, small; 0.33–0.474, moderate; and ≥ 0.474, large (Meissel and Yao 2024; Romano et al. 2006). Positive values indicate higher scores in the BALL group, whereas negative values indicate higher scores in the NO BALL group.

## 3 Results

**FIGURE 1:**
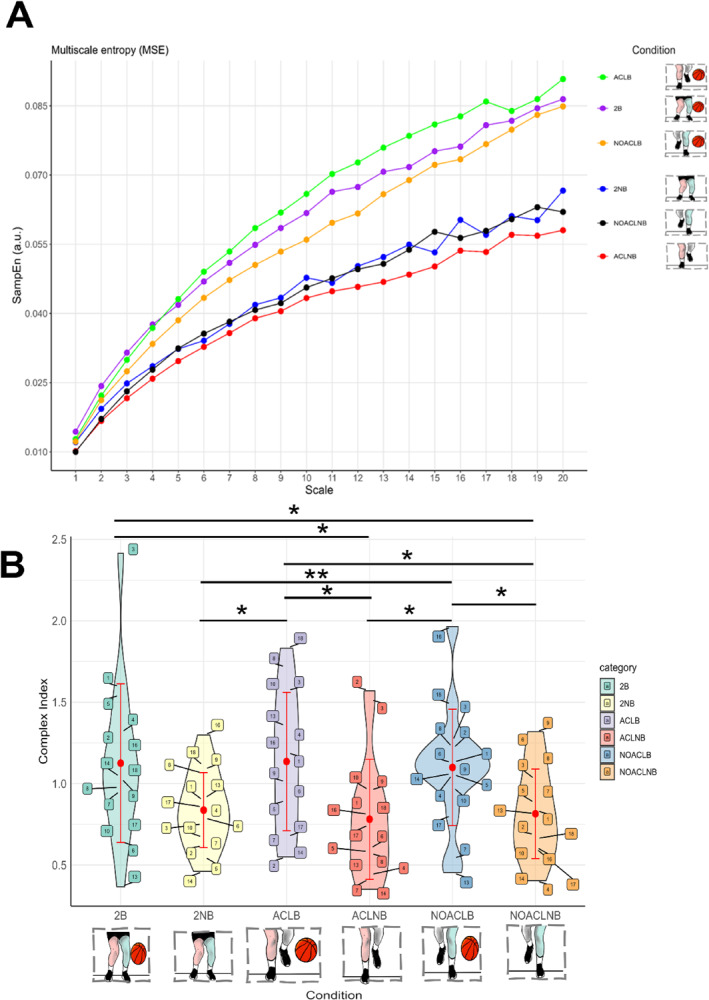
A: Multiscale entropy (MSE); B: Complexity Index (CI); 2NB: Bilateral No Ball; 2B: Bilateral Ball; ACLNB: ACL No Ball; ACLB: ACL Ball; NOACLNB: No ACL No Ball; NOACLB: No ACL Ball (NOACLB); ACL: Anterior Cruciate Ligament; *p < 0.05; **p < 0.01.

**FIGURE 2:**
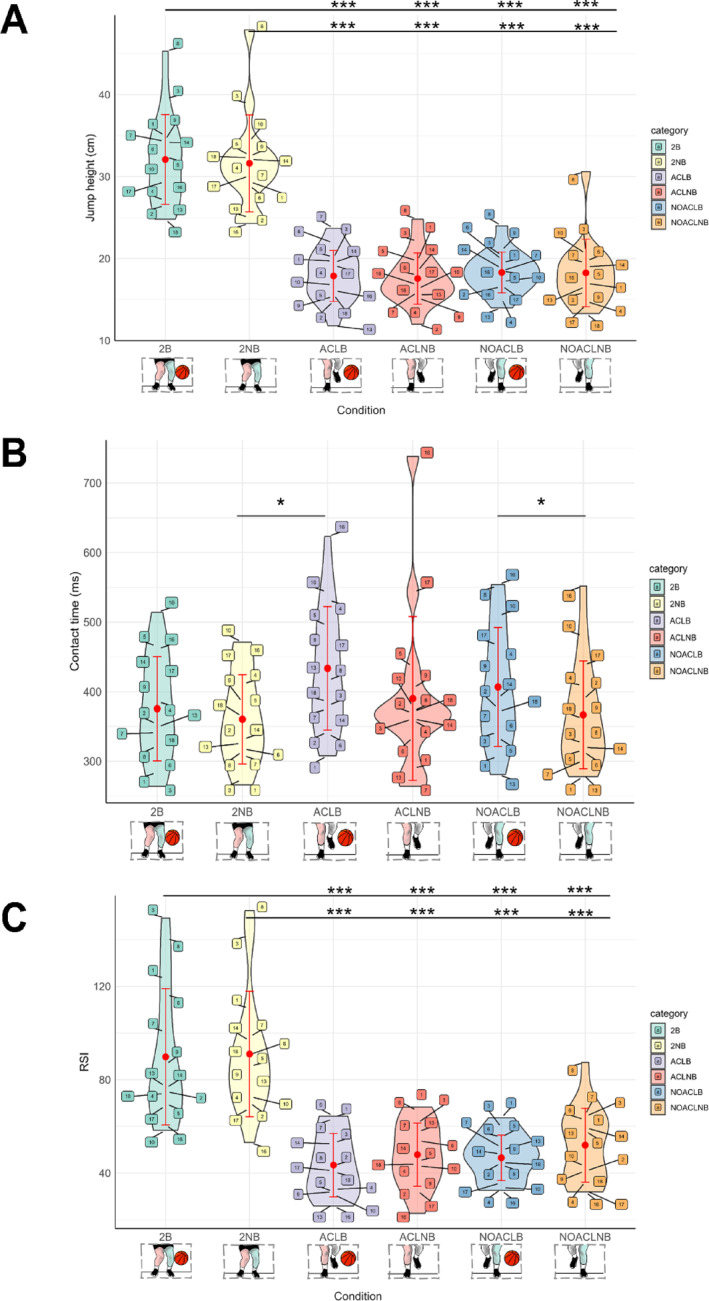
A: Jump height; B: Contact Time (CT); C: Reactive Strength Index (RSI); 2NB: Bilateral No Ball; 2B: Bilateral Ball; ACLNB: ACL No Ball; ACLB: ACL Ball; NOACLNB: No ACL No Ball; NOACLB: No ACL Ball; ACL: Anterior Cruciate Ligament; *p < 0.05; ***p < 0.001.

The results obtained in the present study are presented in Figure 1, 2, 3. Figure 1A illustrates the results of the MSE analysis. An increased trend is shown across all scales in the SampEn values in the ACLB, 2B, and NOACLB conditions, while the ACLNB, 2NB, and NOACLNB conditions showed the lowest values. These data are complemented by the statistical analysis in CI. Figure 1B shows violin plots of the results obtained in CI. Specifically, CI values were significantly higher in ACLB compared to 2NB (*p* = 0.0125), and in 2B compared to ACLNB (*p* = 0.0125). The ACLNB condition also exhibited significantly lower CI values than ACLB (*p* = 0.0125) and NOACLB (*p* = 0.0103), confirming its consistently reduced complexity. Additionally, NOACLB presented significantly higher CI values than 2NB (*p* = 0.0084), while NOACLNB differed significantly from ACLB (*p* = 0.0302) and NOACLB (*p* = 0.0413).

**FIGURE 3:**
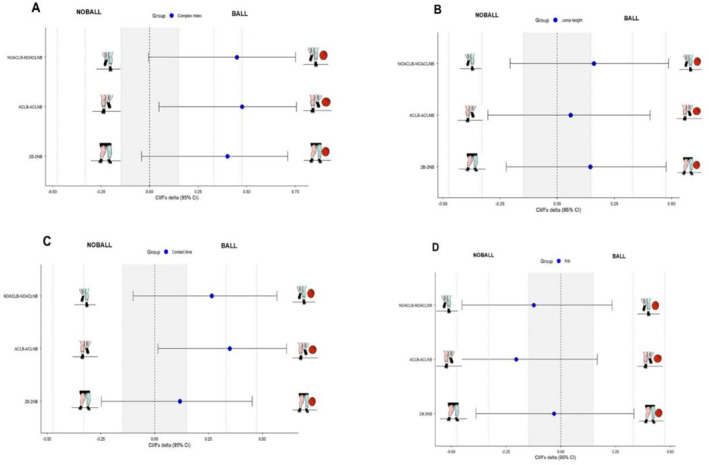
A: Complexity Index (CI); B: Jump height; C: Contact time (CT); D: Reactive Strength Index (RSI); 2NB: Bilateral No Ball; 2B: Bilateral Ball; ACLNB: ACL No Ball; ACLB: ACL Ball; NOACLNB: No ACL No Ball; NOACLB: No ACL Ball; ACL: Anterior Cruciate Ligament.

Figure 2 shows the values of jump height (Figure 2A), CT (Figure 2B) and RSI (Figure 2C) across all conditions. For jump height, significant differences were observed among conditions (*p* < 0.001), mainly between bilateral conditions (2B and 2NB) and unilateral conditions. Jump height in 2B was significantly higher than in ACLB (*p* = 0.00055), ACLNB (*p* = 0.00055), NOACLB (*p* = 0.00055), and NOACLNB (*p* = 0.00055). Similarly, 2NB differed significantly from ACLB (*p* = 0.00580), ACLNB (*p* = 0.00055), NOACLB (*p* = 0.00055), and NOACLNB (*p* = 0.00055), while no significant differences were observed among unilateral conditions. RSI showed a similar pattern, with significant differences between 2B and ACLB, ACLNB, NOACLB, and NOACLNB (all *p* = 0.00092), and between 2NB and the same unilateral conditions (all *p* = 0.00092), again without significant differences among unilateral conditions. Regarding CT, the main significant differences were observed between ACLB and 2NB (*p* = 0.0124), and between NOACLNB and both ACLB (*p* = 0.0092) and NOACLB (*p* = 0.0269), with longer CT values in ACLB and NOACLNB. All statistically significant pairwise differences are represented by lines and asterisks at the top of the graph.

Finally, Figure 3 illustrates the effect size changes associated with the introduction of the ball across all conditions (NOACLB‐NOACLNB; ACLB‐ACLNB; 2B‐2NB). The CI showed moderate to large changes in all comparisons, specifically NOACLB‐NOACLNB (*δ* = 0.45), ACLB‐ACLNB (*δ* = 0.48), and 2B‐2NB (*δ* = 0.40). For jump height, changes were trivial (*δ* < 0.14) in 2B‐2NB and ACLB‐ACLNB and small in NOACLB‐NOACLNB (*δ* = 0.16). Regarding CT, small effects were observed between conditions NOACLB‐NOACLNB (*δ* = 0.26), moderate effects between ACLB‐ACLNB (*δ* = 0.34), and trivial effects for 2B‐2NB (*δ* = 0.12). The RSI showed small effects in ACLB‐ACLNB (*δ* = −0.20) and trivial effects in NOACLB‐NOACLNB and 2B‐2NB.

## 4 Discussion

### 4.1 Main Findings

The findings of the present study provide new insights into MV and neuromuscular control in female basketball players with a history of ACL reconstruction during DVJ. The primary finding is that although linear performance parameters such as jump height, contact time CT, and RSI showed no significant differences between the injured and non‐injured limbs, MV analysis revealed distinct patterns between conditions. Specifically, MSE and CI were highest in the ACLB, 2B and NOACLB condition and lowest in the ACLNB, 2NB and NOACLNB condition. These findings suggest that the previously injured limb, despite demonstrating recovered physical performance, may still present alterations in neuromuscular control strategies. Such patterns are consistent with the Loss of Complexity Hypothesis, which proposes that biological systems under stress or dysfunction tend to exhibit reduced variability and diminished adaptability (Lipsitz et al. 1992). Therefore, while traditional performance metrics may indicate functional symmetry, nonlinear analyses may reveal persistent underlying alterations in motor control following ACL injury.

### 4.2 Nonlinear Parameters Findings

Nonlinear analysis provided further insight into these neuromuscular alterations. In the present study, differences in MSE and CI between injured and non‐injured conditions suggest that neuromuscular control strategies may remain altered following ACL reconstruction, even when linear performance appears symmetrical. Previous studies have reported that reduced nonlinear variability is associated with decreased movement complexity and more rigid motor control patterns in individuals following ACL injury (Georgoulis et al. 2006; Terada et al. 2015; Zampeli et al. 2010). These findings align with the Loss of Complexity Hypothesis, which states that healthy biological systems exhibit complex and adaptable patterns of variability, whereas injury or dysfunction can lead to more constrained and predictable movement behaviors (Lipsitz et al. 1992). Several mechanisms may contribute to these alterations, including impaired proprioception, reduced neuromuscular coordination, and greater reliance on feedforward motor control strategies following ligament injury (Gokeler et al. 2019). In addition, arthrogenic muscle inhibition after ACL reconstruction may restrict movement patterns due to altered mechanoreceptor function and disrupted sensory feedback (Kiefer et al. 2013; Rice et al. 2014; Rice and McNair 2010). In this regard, Chaney et al. (2023) reported neuromuscular changes following ACL reconstruction using Shannon entropy measures, while, Paterno et al. (2015) observed progressive reductions in movement complexity and increased system predictability following subsequent injuries. Collectively, these findings suggest that ACL injury may lead to long‐term alterations in the neuromuscular system's capacity to generate adaptable and efficient motor behaviors.

### 4.3 Linear Parameters Findings

In contrast to the nonlinear findings, the present study observed no significant differences in traditional performance metrics, including jump height, RSI, and CT, between injured and non‐injured limbs. These results are consistent with previous research reporting symmetrical jump performance following ACL reconstruction (Gokeler et al. 2017; Paterno et al. 2018). For example, Gokeler et al. (2017) reported that ACL‐reconstructed athletes were able to achieve similar jump heights between limbs under standardized testing conditions despite persistent neuromuscular deficits. Similarly, Paterno et al. (2018) highlighted that although conventional hop and jump tests may indicate apparent functional recovery, underlying biomechanical asymmetries and neuromuscular deficits can remain present. One explanation for these findings is that athletes may adopt compensatory movement strategies that allow them to achieve similar performance outcomes while masking underlying neuromuscular deficits. Consequently, reliance solely on linear performance indicators may provide an incomplete representation of neuromuscular readiness for RTP. These findings reinforce the need to complement traditional performance metrics with more sensitive analytical approaches capable of detecting subtle alterations in motor control.

### 4.4 Influence of Limb Dominance

In the present study, 93.33% of participants were right‐leg dominant, and most ACL reconstructions were performed on the dominant limb, so observed MV patterns should be interpreted in light of limb dominance. Retrospective studies have shown that leg dominance does not significantly affect short‐term functional outcomes or strength recovery after ACL reconstruction (Boo et al. 2020; Suh et al. 2021). However, differences may emerge in parameters related to motor control and dynamic balance. Indeed, Xiao et al. (2026) reported superior dynamic balance recovery when the dominant limb was injured, compared with the non‐dominant leg. In unilateral tasks such as DVJ, which require high motor control and dynamic balance, recovery may occur earlier in the dominant limb, suggesting that MV could normalize faster with an intervention program. This interpretation should be approached with caution, as longitudinal studies specifically evaluating MV over time are lacking.

### 4.5 Task‐Related Differences

An additional key finding of this study relates to the influence of task constraints on neuromuscular variability. Nonlinear differences in MV were more pronounced in the ACLB condition, suggesting that the inclusion of an external task constraint, such as catching a ball, may accentuate neuromuscular control differences between conditions. Introducing an additional motor–cognitive demand appeared to increase variability in the injured limb, potentially reflecting altered adaptability when athletes are exposed to more complex task environments. This response can be interpreted within the framework of Optimal Variability Theory, which proposes that both excessively low and excessively high variability may indicate suboptimal motor control depending on the context and task demands (Stergiou et al. 2006). In this context, increased entropy and complexity during ball‐included conditions may reflect adaptive flexibility rather than neuromuscular dysfunction. It is also consistent with Newell's Model of Motor Variability, which suggests that movement variability emerges from the dynamic interaction between the individual, the task, and the environment (Newell and Vaillancourt 2001). In sport‐specific contexts such as basketball, where athletes must integrate perceptual and motor information under time constraints, higher levels of variability may reflect the capacity to adapt to unpredictable environmental demands (Exel et al. 2019; Moras et al. 2018; Fernández‐Valdés Villa 2020).

### 4.6 Dual Task Assessments

Previous studies have emphasized the importance of incorporating dual‐task and sport‐specific challenges into RTP assessments because they better reflect real‐game demands (Nordin and Dufek 2019). Therefore, evaluating athletes under more ecologically valid conditions may reveal neuromuscular deficits that remain undetected during standardized testing protocols (Almonroeder et al. 2018). The findings of this study have important implications for RTP assessment and rehabilitation strategies following ACL injury. Traditional RTP evaluations often rely on linear performance measures such as jump height or hop distance, which may suggest complete functional recovery despite persistent neuromuscular alterations. The present results highlight the potential value of incorporating nonlinear analytical methods, such as MSE and CI, to better capture the complexity and adaptability of neuromuscular control (Bittencourt et al. 2016). Rehabilitation programs should therefore prioritize not only restoring performance symmetry but also promoting adaptable motor control strategies capable of responding to dynamic sport‐specific demands. Previous research suggests that perturbation‐based training, variable task constraints, and sensorimotor challenges may help restore MV and enhance neuromuscular adaptability (Davids et al. 2003; Fernández‐Valdés et al. 2020; González‐Millán et al. 2024; Moras et al. 2018; Stergiou et al. 2006; Tuyà Viñas, Fernández‐Valdés Villa, Pérez‐Chirinos Buxadé, González, et al. 2023). Incorporating these approaches into RTP protocols, alongside nonlinear assessment metrics, may improve the detection of residual neuromuscular deficits and ultimately contribute to reducing reinjury risk in athletes returning to sport after ACL reconstruction.

### 4.7 Limitations

While this study provides novel insights, several limitations should be acknowledged. First, the sample size was relatively small, limiting generalizability; however, the sample is highly specific, comprising female basketball players with at least one ACL reconstruction. Although the sample size was sufficient for the detection of moderate‐to‐large effects (power = 0.938), the heterogeneity in surgical characteristics (e.g., graft type, time since surgery, concomitant injuries) and the predominance of dominant‐leg reconstructions may have introduced variability in MV outcomes. This could partially account for differences in movement strategies observed between unilateral conditions. The role of dominance, in particular, deserves further exploration, as habitual motor preferences may interact with compensatory neuromuscular control after ACL injury. While statistical stratification was not feasible due to the sample size, future studies with larger cohorts should address this explicitly. Secondly, only DVJ performance was assessed, whereas tasks such as cutting or lateral movements may provide additional insight into neuromuscular recovery. Third, variability existed in time elapsed between injury and RTP, as well as between RTP and testing. Finally, no Bonferroni or other corrections for multiple comparisons were applied in the post hoc analysis, increasing the risk of Type I error. These findings should therefore be considered exploratory and interpreted accordingly, although the inclusion of Cliff's delta effect sizes supports interpretation of effect magnitude.

It should be noted that, although the ball‐catching task introduces an ecologically valid dual‐task constraint, in the present study did not include direct measures of cognitive or attentional load, such as reaction time, subjective workload (e.g., NASA‐TLX), or neurocognitive monitoring. This represents a limitation, as increased MV in dual‐task conditions could stem from multiple sources, including adaptive motor reorganization, but also potentially uncontrolled variability due to attentional disruption or task interference (Almonroeder et al. 2018; Nordin and Dufek 2019). Dual‐task paradigms have shown that ACL‐reconstructed athletes exhibit altered neuromechanics under cognitive load, which can either enhance or degrade performance depending on attentional capacity and motor automatization (Monfort et al. 2022; Shi et al. 2021). Our results may thus reflect a combination of increased perceptual‐motor integration demands and cognitive stress, both of which affect neuromuscular control. Future studies should aim to quantify cognitive demands during dual‐task assessments to more accurately interpret nonlinear variability outcomes and their relation to neuromuscular adaptability versus instability.

## 5 Conclusion

The inclusion of a task constraint did not result in significant differences between ACL and NOACL groups in linear biomechanical variables related to jumping performance, including jump height, RSI, and CT. In contrast, nonlinear analyses of MV using MSE and CI revealed meaningful differences between conditions, with moderate‐to‐large effect sizes (*δ* > 0.4). Lower entropy and complexity were observed in the no‐ball condition, whereas higher values emerged when the task constraint (ball) was introduced.

These findings highlight that, despite apparently symmetrical physical performance, the reconstructed limb may continue to exhibit neuromuscular rigidity in standard conditions. The observed increase in complexity under dual‐task constraints suggests that incorporating sport‐specific challenges—such as ball handling or reactive elements—can uncover hidden deficits in motor adaptability. Practically, this underscores the importance of including nonlinear assessments and dual‐task scenarios in return‐to‐play protocols to better evaluate neuromuscular readiness and reduce reinjury risk.

This study highlights the limitations of relying solely on linear performance metrics in post‐ACL return‐to‐play (RTP) decision‐making and emphasizes the value of nonlinear analyses for identifying persistent neuromuscular deficits. RTP programs should incorporate variability‐based training approaches to promote adaptive motor control, and RTP evaluations should include task‐specific assessments that challenge cognitive–motor demands.

## Author Contributions

S.G.‐M., T.C. and B.F.‐V: conceptualization and methodology. S.G‐M., C.P.‐C.B., B.F.‐V. and V.I.‐D.: formal analysis. S.G.‐M., C.P.‐C.B., V.T‐R., V.I.‐D., L.A.‐A. and B.F.‐V.: investigation. S.G.‐M., C.P‐C.B., G.M, and B.F.‐V.: data curation. S.G.‐M., V.T‐R, T.C. and B.F.‐V.: writing – original draft preparation. V.I.‐D., L.A.‐A., T.C., G.M. and C.P.‐C.B: writing – review and editing. T.C. and B.F.‐V.: supervision. All authors have read and agreed to the published version of the manuscript.

## Funding

The present study was supported by TecnoCampus of the Universitat Pompeu Fabra and by Research group in Technology Applied to High Performance and Health (TAARS) (33/2023). Víctor Toro‐Román is the recipient of a postdoctoral grant from the Pla de recuperació, transformació i resiliència—finançat per al Unió Europea—Next Generation EU (200015ID3).

## Ethics Statement

The study was approved by Ethics Committee for Clinical Research of the Catalan Sports Council (026/CEICGC/2021).

## Conflicts of Interest

The authors declare no conflicts of interest.

## Data Availability

The original contributions presented in the study are included in the article, further inquiries can be directed to the corresponding author.
